# Associations Among Stressful Events, Social Support, and Alcohol Use in Women and Men

**DOI:** 10.3390/bs16020261

**Published:** 2026-02-11

**Authors:** Ani Hovnanyan, Rajita Sinha, Nia Fogelman

**Affiliations:** 1 Department of Psychiatry, Yale School of Medicine, New Haven, CT 06511, USA; 2 Yale Stress Center, Yale School of Medicine, New Haven, CT 06511, USA; 3 Department of Developmental Psychology and Socialization, University of Padova, 35128 Padua, Italy

**Keywords:** stress amount and intensity, alcohol use, social support, protective factors, sex differences

## Abstract

Recent stressors may increase the risk of alcohol misuse. However, the number and duration of recent stress, whether social support (SS) moderates these effects, and whether this differs for men and women, are unclear. This cross-sectional study examined the effects of these factors on alcohol use severity, amount, and frequency in 462 community adult women and men. Linear regression (controlling for sex, age, and education) indicated that more stressful life events and longer stress duration were associated with a greater probability of any alcohol use and greater alcohol use severity as measured by the Alcohol Use Disorder Identification Test (AUDIT). More stressful events were associated with a greater amount of alcohol consumption. Longer stress duration also interacted with sex and SS to predict AUDIT scores, such that high SS, only for men, predicted a higher AUDIT score, but higher stress duration predicted AUDIT scores for women, regardless of SS score. More stress events with high social support predicted a greater alcohol use amount, only in men. Current findings demonstrate that significant impacts of the number and duration of recent stressors increase the risk of alcohol intake and severity. Furthermore, SS uniquely promotes drinking in men, suggesting male-specific increased alcohol risk. Future work would benefit from further disentangling whether these effects stem from certain types of SS (i.e., emotional, financial, practical) or if these effects were due to the nature of the social interactions (i.e., drinking buddies). Moreover, future work should continue to explore the multifaceted nature of stress as well as consider how sex and SS impact alcohol use.

## 1. Introduction

Excessive alcohol consumption has adverse social, psychological, and medical consequences, including increased risk for mortality (Cui et al., 2025; Kranzler, 2023). While there are many motivations to drink, people frequently consume alcohol to dampen stress and negative affect. Many social and problem drinkers expect alcohol to relieve tension and stress-related negative emotions (Willis et al., 2025). The link between stress and alcohol consumption has been systematically studied. Jose et al. (2000) showed that people consume more alcohol during and after stressful life events. Those who misuse alcohol report more stressful life events as compared to non-abusers (Kirsch et al., 2024; Zhou et al., 2025). Stress-motivated alcohol consumption is a matter of concern since it can lead to alcohol use disorder (Sinha, 2008, 2012). Stressful events are experienced at different severities, and the severity of stress may be related to the level of alcohol consumption (Smith et al., 2018). Even though stress-related drinking is well studied, less is known about the effects of different aspects of the stressor, such as duration. Systematic research on these aspects is needed.

People face stressful and traumatic events across the lifespan. The effects of recent versus past stressors on health behaviors may differ; events occurring recently (i.e., within the past 12 months) are more likely to be related to behaviors like binge drinking, relative to events that occurred prior to the past 12 months (Hermes et al., 2021). Existing data emphasize the impact of recent stressors on drinking and show that stressor-related drinking may lead to future alcohol problems (Russell et al., 2017). Therefore, it is important to better study the relationship between exposure to recent stressors and alcohol use, especially targeting potential protective factors to foster timely interventions. Furthermore, stressful events may occur over a short or long period of time. For example, the duration of an event, such as natural disasters or serious accidents, is time-limited. In contrast, effects of violence, loss and grief, financial difficulties, and relationship issues may potentially have a long duration (Gottlieb, 2013). Stressful events that last longer have been associated with adverse mental health outcomes, such as a more severe presentation of post-traumatic stress disorder (Buydens-Branchey et al., 1990) and increased likelihood of maladaptive behavioral responses (Wilson, 2010). Thus, studying the effect of stressful event duration could be of potential relevance in understanding risky alcohol consumption. While existing evidence on stress and alcohol use is mainly focused on the number of stressful events (e.g., Slopen et al., 2011) and the event type (e.g., Shaw et al., 2011), we lack knowledge on stressor duration effects on alcohol use quantity, frequency, and severity.

In contrast to drinking in response to stress, which is considered a maladaptive coping response (Sinha, 2008), social support (SS) has long been associated with buffering stress and regarded as an adaptive coping strategy (Acoba, 2024; Hinz et al., 2025). SS is characterized as people’s perception or experience of being cared for by others and having a reliable network to turn to in times of need (Taylor, 2011). In addition, having high SS may be protective against problematic alcohol drinking (Jodis et al., 2023), and a lack of SS may facilitate alcohol use (Borsari & Carey, 2006). The protective, stress buffering effects of SS have been well documented. For instance, Steptoe et al. (1996) showed that a larger supportive network led to a lower amount of alcohol use during stress. Recent data shows that SS decreased stress-motivated alcohol drinking in US Navy members (Kelley et al., 2017). However, the role of SS in alcohol consumption may be more nuanced. For example, having close friends living nearby may decrease alcohol drinking, whereas socializing with neighbors may increase it (Platt et al., 2010). Parental support was found to be associated with fewer symptoms of alcohol misuse and dependence, but peer support was related to more symptoms in adolescents (e.g., Urberg et al., 2005). To date, the role of SS in stress-related drinking has not been systematically studied, and the focus has been on the number of events and type (see Jennison, 1992), while studies on stressful event duration are rare.

The relationship between stress and alcohol use may also vary by sex, and the impact of SS on the stress–drinking relationship may be different depending on the type of SS being received by women and men. Namely, women report higher rates of traumatic and stressful events (Guinle & Sinha, 2020) and related negative mental health outcomes (Olff, 2017), potentially representing one pathway to problematic drinking that may be more common in women than men (Guinle & Sinha, 2020). On the other hand, stronger effects of stressful life events on alcohol use have been reported in men compared to women (Veenstra et al., 2006). Moreover, recent evidence shows that sex can moderate stress responses in individuals who have unhealthy alcohol use (Moreno-Fernández et al., 2024). Therefore, sex may be an important moderator of the stress and alcohol use relationship (Patock-Peckham et al., 2022). Additionally, the type of social networks from which women and men receive SS may vary (Borsari & Carey, 2001; Homish & Leonard, 2008). Previous research found that insufficient social support is linked to problematic drinking in women as compared to men (Maxwell et al., 2022). Overall, these findings emphasize the importance of studying sex differences in the potential effect of SS on stress-motivated alcohol use.

While stress is associated with increased problematic alcohol consumption, both the number and duration of stressful life events have not been thoroughly explored. Furthermore, SS and sex may moderate the relationship between stress and alcohol consumption. Therefore, we recruited a community sample to assess the effect of recent stressful events on alcohol use. We measured the number and total duration of recent stressors and considered the potential moderating effect of SS and sex differences. Our primary research question asked whether the number and duration of recent stressful events were associated with any weekly use of alcohol, as measured in binary (yes/no) and problematic alcohol use, as measured by the AUDIT. We chose to focus on recent stressors that occurred within the past 12 months, given their specific relationship previously identified with unhealthy alcohol use (see Hermes et al., 2021). We studied severity (AUDIT), any alcohol use, amount, and frequency of alcohol use to capture integrated aspects of alcohol consumption, as these measures reflect distinct drinking behaviors and potential risks, providing a more comprehensive picture of the links between stress, alcohol and social support. Consistent with previous findings (Blaine & Sinha, 2017; Buydens-Branchey et al., 1990), we expected that more stressful events and longer stress duration would be associated with higher rates of alcohol use and greater problematic alcohol use. We also expected that a higher amount of stress and longer stress duration together with low SS would be associated with higher alcohol use rates (Kelley et al., 2017), specifically in women compared to men (Maxwell et al., 2022). Based on this previous research, we hypothesized that both any alcohol use and problematic use would be associated with greater drinking, but that problematic use would be influenced by both SS and sex. Secondly, if sex moderated the influence of SS on the relationship between stress and problematic drinking, we planned to examine this relationship separately in women and men.

## 2. Materials and Methods

### 2.1. Participants

A community sample of 462 (220 women; 242 men) in New Haven, Connecticut, between the ages of 18 and 61 (M = 29.53, SD = 9.07) were enrolled to assess recent stressful life events and current alcohol use in this cross-sectional study. Participants were recruited through online advertisements on social media, in local newspapers, and at community centers between 2008 and 2012, as previously described (e.g., Xu et al., 2018; Hovnanyan, 2023). Participants were recruited to be part of this cross-sectional study, with the opportunity to participate in subsequent Yale Stress Center studies. They were included in the study if they were able to read at the sixth-grade level. Exclusion criteria were as follows: acute current psychiatric illnesses requiring medical attention, lifetime opioid use disorders or other substance use disorders (excluding nicotine, alcohol, and cannabis) and any current or past use of opiates, use of prescribed medications for any psychiatric disorders, pregnancy, chronic medical conditions, and traumatic brain injury or loss of consciousness. These exclusion criteria were used to ensure the sample was generally healthy and a representative community sample. All the participants provided informed consent to participate, and the protocol was approved by the IRB of Yale.

### 2.2. Procedures

To determine eligibility, potential participants completed an initial telephone screening. Participants were then invited to the Yale Stress Center for an in-person assessment, where they signed written informed consent and completed well-validated surveys and structured interviews on demographics, stressful life events, SS, and alcohol use.

#### 2.2.1. Recent Stressful Events

The Recent Life Events subscale of the Cumulative Adversity Index (CAI; adapted from Turner & Lloyd, 1995) was used to measure participants’ exposure to recent stressors (Hermes et al., 2021; Ansell et al., 2012). The CAI is a widely used 140-item semi-structured interview. The 34-item Recent Life Events subscale asks (1) if an event happened to themselves or a close loved one in the past 12 months and (2) when such an event began and ended. Events are related to death and loss of loved ones, violence, natural disasters, serious accidents and injuries, financial difficulties, issues related to relationships, marital status, work, and education. The internal reliability of the Recent Life Events subscale was consistent with other studies, and overall, previous research reported high reliability of CAI and its subscales (e.g., Ansell et al., 2012). In this sample, the reliability of CAI was high (Cronbach’s α = 0.88), and the Recent Life Events subscale had a Cronbach’s α = 0.65, reflecting the diverse variety of adverse life events captured in the measure.

To obtain the Recent Life Events total score (stress amount), the sum of the number of endorsed items was calculated, ranging from 0 to 34. To calculate the event duration (stress intensity), the time period of exposure of the event was determined by the sum of the difference between the end and start dates. Then the score was averaged by dividing the sum score by the number of endorsed items for each participant. The final duration score ranged from 0 (no event) to 4, where single 1-day events were coded as 1, events lasting from 1 to 6 and 6–12 months were coded as 2 and 3, respectively, and the events lasting more than 12 months were coded as 4 (Buydens-Branchey et al., 1990).

#### 2.2.2. Social Support

To assess participants’ perceived SS, the Interpersonal Support Evaluation List (ISEL; Cohen & Hoberman, 1983) was used. This widely used questionnaire measures an individual’s perception of SS from others at stressful times and in general. It is a 40-item, dichotomous (true/false) scale. The total score of the ISEL is calculated by summing up all the items. The ISEL had a Cronbach’s α = 0.87 for this study.

#### 2.2.3. Alcohol Use

Participants were asked to report on whether they consumed any alcohol as defined by at least monthly or more frequent use patterns, by answering a dichotomous (yes/no) question. In addition, the amount and frequency of alcohol use during the last month were assessed by responses to two items inquiring about the typical number of drinks consumed per drinking occasion and the number of days they drank alcohol in the past 30 days.

To measure the alcohol use severity, the Alcohol Use Disorders Identification Test (AUDIT; Saunders et al., 1993) was used. The AUDIT is a 10-item frequently used instrument assessing the severity of drinking and hazardous alcohol use as well as the psychological impact of drinking (e.g., feelings of guilt and difficulties in remembering things). The total AUDIT score is obtained by summing up the scores of each item, which range from 0 to 40. The total AUDIT score had a Cronbach’s α = 0.89 for our sample.

### 2.3. Data Analysis

R v. 4.0.3/RStudio v. 2026.01.0+392 (RStudio Team, 2021) was used for the data organization and analysis in this study. Demographic data was presented for the overall sample and tested for sex differences using *t*-tests for continuous variables and chi-square tests for categorical ones. Correlation analysis was then performed to measure the relationship between stress, alcohol use, SS, and demographic variables.

Linear regression models were performed to examine whether recent life stressors predicted alcohol use, including (1) the presence of any alcohol use in the past 30 days (yes/no; logistic regression), (2) alcohol misuse severity (AUDIT), and alcohol amount in the past month. The number and duration of recent life stress by SS by sex models were assessed for each outcome measure. One main model assessed stress (amount or duration, in separate models) by sex by SS interactions for alcohol variables, and this model was applied to each alcohol outcome. The number and duration of stress by SS models were assessed for alcohol use amount and frequency separately for women and men. Age and education were included as covariates. When the interaction effect reached significance, simple slopes of the associations were examined. To address the multicollinearity issue in all models, stress and SS variables were mean-centered. Square-root-transformed dependent variables (AUDIT score) were used to correct for abnormal residuals (i.e., non-constant variance).

## 3. Results

Our total sample consisted of 462 adults (with a mean age of 29.53 (SD = 9.07); 47.6% female). This sample was predominantly Caucasian (63.4%) and displayed moderate-risk alcohol consumption levels (AUDIT score mean = 7.11, SD = 7.44). Men and women did not differ significantly on any demographic, stress, or SS variables (*p*’s > 0.518). However, and as expected, women had significantly lower drinking problem scores and less alcohol use, and amount and frequency of alcohol consumption in the past month (*p*’s < 0.001). Complete descriptive information can be found in Table 1.

Correlation analysis showed positive relationships between stress and alcohol use variables, with the number of recent life events related to a greater amount and severity of alcohol use for women (*p*’s < 0.05) and severity for men (*p* < 0.05), and stress duration was associated with alcohol use severity for women (*p* < 0.05) and for men (*p* < 0.05). SS was negatively related to stress and alcohol amount and severity for women (*p*’s < 0.01) and for men (*p*’s < 0.05). A detailed description of the correlation results can be found in Table 2.

Regression models assessing stress (number and duration), SS, and sex effects are found in the sections below. Table 3 provides an overview of regression model findings, including significant and non-significant terms.

### 3.1. Duration of Stressful Events Predicts Any Alcohol Use

**Figure 1:**
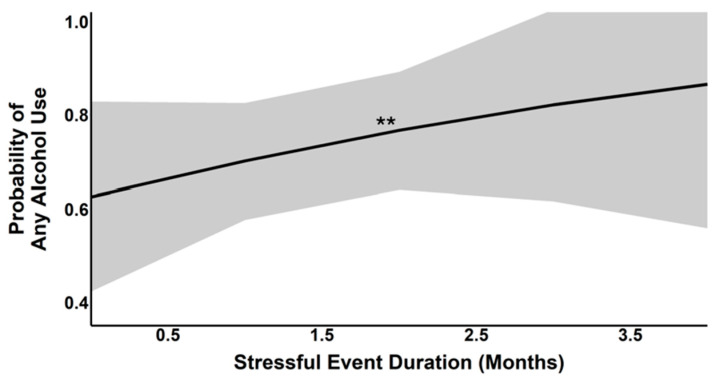
Association between the duration of stressful events (*x*-axis, measured in months) and the predicted probability of any alcohol use (*y*-axis, ranging from 0 to 1). The black regression line indicates a positive relationship, suggesting that a longer duration of stressful events is associated with an increased likelihood of any alcohol use. Shading around the line indicates standard error of the estimate. ** *p* < 0.01.

Regression models assessing the effects of stress (number and duration), SS, sex, and their interactions with the prevalence of any alcohol use were conducted. Findings demonstrated that longer stress duration was associated with a greater probability of any alcohol use (*X*^2^(1) = 9.00, *p* < 0.003; see Figure 1), but the number of stressful events was not (*X*^2^(1) = 1.47, *p* > 0.225). Moreover, men had a greater probability of any alcohol use as compared to women (model with event number: *X*^2^(1) = 6.48, *p* < 0.01; model with event duration: *X^2^*(1) = 7.61, *p* < 0.001). Higher age was associated with a greater probability of any alcohol use (model with event number: *X*^2^(1) = 6.21, *p* < 0.01; model with event duration: *X*^2^(1) = 5.19, *p* < 0.02). No other higher-order interactions were significant.

### 3.2. Number and Duration of Recent Stressful Events Predict Alcohol Use Severity (AUDIT Scores) with Social Support Moderation for Men

Models testing stress by SS by sex on alcohol use severity (AUDIT) indicated a main effect of number and duration of stressors on AUDIT scores (number of stressors: *F*(1, 451) = 19.50, *p* < 0.001; duration of stressors: *F*(1, 451) = 10.45, *p* < 0.002). Specifically, more stressful events and a longer stress duration were associated with higher AUDIT scores (number of stressors: *B* = 0.11, *t* = 4.42, *p* < 0.001; duration of stressors: *B* = 0.20, *t* = 3.24, *p* < 0.002). Men had higher AUDIT scores as compared to women (model with event number: *B* = 0.69, *t* = 5.69, *p* < 0.001; model with event duration: *B* = 0.69, *t* = 5.67, *p* < 0.001). Longer stress duration interacted with SS and sex to predict the AUDIT score (*F*(1, 451) = 5.88, *p* < 0.016), such that SS did not significantly moderate the effects of longer event duration on AUDIT scores for women (*p* > 0.32). However, this effect was significant for men (*B* = −0.07, *t* = −2.53, *p* < 0.02). Covariates (age and education) and other interaction terms were not statistically significant in these models.

**Figure 2:**
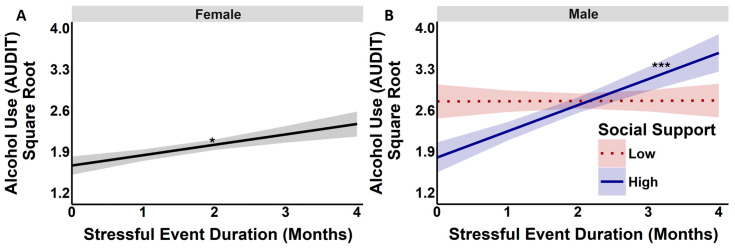
Association of levels of stressful event duration predicting alcohol use (AUDIT) in sex-specific models. (**Panel A**) Longer event duration predicted a higher AUDIT score for women (*p* < 0.032). (**Panel B**) For men, the impact of event duration on the AUDIT score was moderated by social support (*p* < 0.018). At high SS (blue line) levels, longer event duration predicted higher AUDIT scores. The *x*-axis represents the duration of stressful events, and the *y*-axis represents the AUDIT score. * *p* < 0.05; *** *p* < 0.001.

To better understand this effect, simple effect models examining stress x SS interactions on AUDIT scores were completed for men and women separately. For both men and women, a main effect of the number of stress events on AUDIT scores was present (women: *F*(1, 214) = 13.50, *p* < 0.001; men: *F*(1, 235) = 7.03, *p* < 0.009). This main effect persisted for women with the duration of stressors (*F*(1, 214) = 4.67, *p* < 0.032). Whereas, unique to men, SS moderated the stress–AUDIT relationship (*F*(1, 235) = 5.76, *p* < 0.018), such that high SS coupled with a longer duration of stressors predicted higher AUDIT scores (*B* = 0.44, *t* = 2.40, *p* < 0.018). See stress-duration-specific models for each sex in Figure 2A,B.

### 3.3. Number and Duration of Stressful Events Interact with Social Support to Predict Alcohol Use

**Figure 3:**
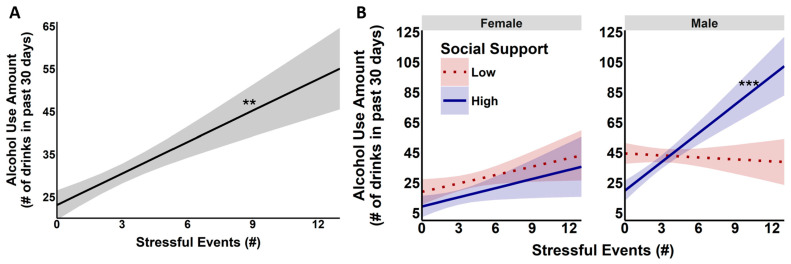
(**Panel A**) Association between the number of stressful events (*x*-axis) and alcohol use amount (*y*-axis). The black regression line highlights the positive association, indicating that an increase in the number of stressful events is related to an increase in alcohol use. (**Panel B**) More SS heightens the impact of stressful events (*x*-axis) to predict a greater amount of alcohol use (*y*-axis) in men only. ** *p* < 0.01; *** *p* < 0.001. # indicates quantity (number).

Regression models assessing effects of stress (number and duration), SS, sex, and their interactions predicting alcohol use amount in the past month showed a main effect of the number of stressful events on the amount of alcohol use (*F*(1, 437) = 7.23, *p* < 0.008). Specifically, as shown in Figure 3A, a higher number of stressful events was associated with a higher quantity of alcohol use (*B* = 2.46, *t* = 2.69, *p* < 0.008). The number of stressful events interacted with SS (*F*(1, 437) = 4.20, *p* < 0.042) such that more stressful events in combination with high SS predicted the greatest quantity of alcohol use (*B* = 0.27, *t* = 2.05, *p* < 0.042). However, this was better understood by a trending SS and sex interaction with the number of stressful events to predict the amount of alcohol use (*F*(1, 437) = 3.81, *p* < 0.052), such that more stressful events with high SS in men predicted a higher amount of alcohol use (*B* = 6.40, *t* = 3.45, *p* = 0.001), but there was no significant relationship in women (see Figure 3B). No other interactions were significant.

**Figure 4:**
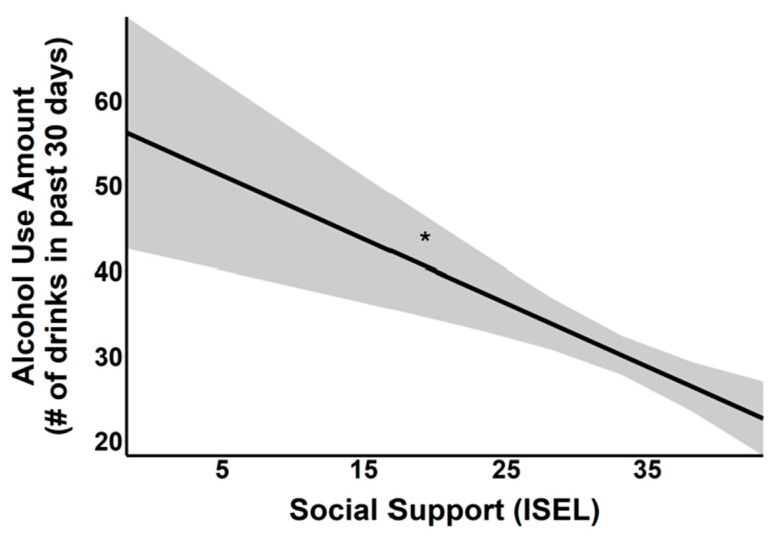
Association between SS (*x*-axis, measured in months) and alcohol use amount (*y*-axis). The black regression line indicates a negative relationship, suggesting that less SS is associated with an increase in alcohol use. Shading around the line indicates standard error of the estimate. * *p* < 0.05. # indicates quantity (number).

In the model assessing the effects of stress duration, there was a main effect of SS on alcohol use amount (*F*(1, 437) = 3.88, *p* < 0.05). Specifically, lower SS was associated with a higher amount of alcohol use (*B* = −0.75, *t* = −1.97, *p* < 0.05; see Figure 4). Women had a lower amount of alcohol use as compared to men (model with event number: *B* = −20.9, *t* = −4.53, *p* < 0.001; model with event duration: *B* = −20.2, *t* = −4.37, *p* < 0.001). Higher age was associated with greater amount of alcohol use (model with event number: *B* = 0.52, *t* = 2.03, *p* < 0.044; model with event duration: *B* = 0.52, *t* = 2.0, *p* < 0.047). No interaction terms were significant.

## 4. Discussion

The current cross-sectional study in a large community sample provides several novel findings on the relationships between stress and problematic alcohol intake. First, consistent with prior research, we found that a greater number of stressful events were associated with greater severity of problematic alcohol use and a greater amount of alcohol consumption (Blaine & Sinha, 2017). Also as expected, longer stress duration was linked to higher alcohol use severity and higher prevalence of any alcohol use. Lower SS predicted greater amounts of alcohol intake. These findings indicate that the exposure to both a high number of stressful events and long-lasting stressors as well as overall low SS contribute to developing problematic drinking behaviors among adults.

However, importantly, the above relationships between stress and problematic alcohol use severity were moderated by SS and sex. Specifically, while women demonstrated a positive relationship between higher stress and greater alcohol use severity regardless of SS, men with high SS showed a stronger positive relationship between stress (duration of recent events) and AUDIT score. Such findings are consistent with the larger literature reporting a positive association between stress amount and alcohol consumption, with SS prompting alcohol use in stress-exposed men (Blaine & Sinha, 2017; Maxwell et al., 2022; Rapier et al., 2019; Platt et al., 2010; Urberg et al., 2005). Moreover, our findings go beyond the well-studied aspects of stress (i.e., number of events) and suggest that SS moderates stress-motivated drinking when people are exposed to not only a higher number of stressors but also to a longer duration of stressful events. Importantly, we showed that both the number and duration of stressful events interacted with SS, and a trending interaction with sex, to predict alcohol use amount. Our results revealed that higher SS paired with more stress events predicted a greater quantity of alcohol use driven by men only.

As sex interacted with stress measures and SS on AUDIT scores, we conducted separate regression models for each sex, which indicated that, among men only, a longer duration of stressful events with high SS predicted greater alcohol symptoms, but not in women. Consistently, prior research has reported that the lack of SS may be related to problematic drinking in women as compared to men (Maxwell et al., 2022). On the other hand, Rapier et al. (2019) found that male prisoners are likely to consume more alcohol when having high levels of SS. Our findings further extend this previous work by directly testing the effects in both men and women, with clear evidence of an opposite effect of SS on the relationship between stress and alcohol use amount and severity in men versus women.

There can be several plausible interpretations for the observed sex-specific effect of SS on the stress–alcohol relationship. One possible explanation may be related to the source and type of SS in women and in men. It is hypothesized that women and men may have different social networks. Namely, men are more likely to have “drinking buddies” as compared to women (Borsari & Carey, 2001). And, even though women and men have comparable social network sizes (Westermeyer et al., 2004), the type of network varies by sex; that is, men tend to have friends who are more supportive of drinking and related activities compared to women (Homish & Leonard, 2008). Notably, even when men do not explicitly ask for SS, getting involved in social interactions and bonding with others may still motivate alcohol use, given that gender-specific norms and social expectations may shape drinking behaviors (Martín del campo-Navarro et al., 2024). Evidence suggests that conformity to traditional masculine norms may be related to increased alcohol consumption (Zamboanga et al., 2024). As a drinking-supportive social community is known to strongly influence heavy alcohol use (e.g., Reifman et al., 2006), it is possible that for men, who may be more prone to having drinking buddies, stress events may facilitate SS networks to promote drinking activities. Thus, future work would benefit from examining differences between SS and social interaction, which are not clearly delineated here. Interestingly, women with high SS do not show this tendency to engage in drinking activities during stress. This difference can be attributed to sex-specific coping strategies. Women are more likely to adopt emotional coping strategies, utilizing SS to process emotions and reduce stress through communication and emotional expression (Tamres et al., 2002). Conversely, men use SS for behavioral coping, and alcohol intake is frequently included in behavioral coping strategies for men (Tamres et al., 2002). These distinct coping mechanisms may help explain why high SS during stress was related to increased alcohol consumption in men but not in women. Overall, our findings support the notion that the source of social networks may have a key role in the link between SS and alcohol drinking (e.g., Platt et al., 2010; Urberg et al., 2005). Thus, in addition to studying the role of SS per se on stress-potentiated drinking, future works may consider studying the type of SS men and women receive.

Most notably, this study examined the impact of different aspects of stress on alcohol use, considering the role of SS and sex. While the number of stressful events has been most frequently studied in the context of stress, SS, and alcohol use (see Jennison, 1992), we found an effect of stressful event duration as well. Our results suggest that SS may promote stress-motivated drinking in men when they are exposed to a high number of stressful events or to long-lasting stressors by shedding light on the less studied aspects of stressful events, such as their duration. With this study, we highlight the importance of considering multiple aspects of stress when studying stress and alcohol use links given the strong influence of both stress amount (number of stressful events) and its intensity (duration of stressful events) on health and related behaviors.

This work has several limitations. First, while we measured the types of SS possibly received, we did not measure who provided the support. This study lacks specificity regarding specific types of SS. Future studies may benefit from examining SS subscales when examining the link to alcohol use. Second, even though we utilized widely used and well-validated measures, the data relied on self-reported information, which depends on memory recall that could have biased the results (e.g., quantity of drinks and dates of stressful events that happened). Third, as the study included a community sample with a range of alcohol use and misuse, it is possible that the associations between stress, SS, and alcohol use may vary amongst different levels of drinking history. And the sex differences we found may partly be linked to group differences in alcohol consumption levels and patterns. Fourth, to measure alcohol amount and frequency, we used single-item retrospective self-reports, which may potentially reduce the precision and reliability of these measures. Finally, this study utilized a cross-sectional design, which permitted obtaining several detailed measures on a fairly large sample; however, it did not allow us to make inferences about the long-term effects of stress and SS on alcohol use. Future longitudinal investigations are needed to address these important limitations to provide a greater understanding of the role of SS in influencing the link between stress and alcohol use. It is noteworthy that the data was collected between 2008 and 2012; therefore, different drinking trends among women and men might be expected today. Additionally, psychophysiological responses to stress may vary by age, and the associations between stress drinking and SS may differ across the lifespan (Peterson-Sockwell et al., 2023; Mikneviciute et al., 2023). Thus, our results based on a sample with a mean age of 29 years are unique to this age group.

In summary, the current study contributed to the stress and alcohol use research by revealing that more stressful events and longer stressful event duration were each associated with a higher prevalence of any alcohol use and greater alcohol use severity. Consistent with Patock-Peckham et al. (2022), we suggest larger studies of mixed-method designs to study sex differences to examine the effect of a variety of stress types and durations on problematic drinking. Studying the choice to use alcohol, the severity of problems related to alcohol, and the quantity all represent different aspects of future problematic drinking. Further, SS differentially impacts the stress–alcohol relationship for men and women, such that high SS may promote stress-potentiated drinking in men. To our knowledge, this was the first study to systematically study various aspects of stress (i.e., amount and intensity), SS, and alcohol use in both women and men to address sex differences by providing an integrative data set of different measures of alcohol use. Given the mental and physical health risks related to stress-motivated problematic alcohol consumption and the importance of exploring protective factors against stress-related drinking, our data underscore the need to further study multiple aspects of the nature of stressful events and consider sex and SS for their impact on health behaviors.

## Figures and Tables

**Table 1:** Descriptive statistics of study variables.

	Female N = 220 (47.62%)	Male N = 242 (52.38%)	Whole Sample N = 462
Age	29.49 (9.41)	29.56 (8.78)	29.53 (9.07)
Education	14.91 (2.24)	14.92 (2.27)	14.91 (2.26)
Employment			
Full-time	44 (22.45%)	57 (26.03%)	101 (24.34%)
Part-time	61 (31.12%)	41 (18.72%)	102 (24.58%)
Unemployed	46 (23.47%)	72 (32.88%)	118 (28.43%)
Never Employed	45 (22.96%)	49 (22.37%)	94 (22.65%)
Race			
Caucasian	126 (57.27%)	167 (69.01%)	293 (63.42%)
African American	56 (25.45%)	53 (21.9%)	109 (23.59%)
Hispanic	20 (9.09%)	11 (4.54%)	31 (6.71%)
Asian	8 (3.64%)	8 (3.3%)	16 (3.46%)
Other	10 (4.54%)	3 (1.24%)	13 (2.81%)
Recent Stressful Events (#)	2.93 (2.57)	2.77 (2.56)	2.85 (2.56)
Recent Stressful Events Duration (months ^t^)	1.61 (0.64)	1.66 (0.96)	1.63 (0.99)
Alcohol Use Severity (AUDIT) *	5.37 (6.63)	8.69 (7.80)	7.11 (7.44)
Any Alcohol Use *			
Yes	148 (67.58%)	190 (78.51%)	338 (73.32%)
No	71 (32.42%)	52 (21.49%)	123 (26.68%)
Alcohol Use Amount (# of drinks, 30 days) *	19.98 (41.77)	39.09 (54.55)	29.88 (49.68)
Alcohol Use Frequency (# days, 30 days) *	5.25 (6.47)	9.27 (8.61)	7.36 (7.91)
Social Support (range 2–40)	33.19 (6.31)	33.22 (6.74)	33.21 (6.54)

* Asterisks indicate significantly different groups: AUDIT score—*t* (460) = −4.89, *p* < 0.001; any alcohol use—*X*^2^(1) = 7.02, *p* < 0.001; alcohol amount—*t* (446) = −4.14, *p* < 0.001; alcohol frequency—*t* (460) = −5.62, *p* < 0.001. Continuous variables are presented as M (SD); categorical variables are frequency (%). ^t^ See the Section 2.2.1 for details on variable creation. # indicates quantity (number).

**Table 2:** Correlation matrix of study variables for women (above diagonal) and men (below diagonal).

	1	2	3	4	5	6	7	8
1. Recent Stressful Events #	—	0.46 ***	0.10	0.16 *	0.30 ***	−0.24 ***	0.07	−0.16 *
2. Recent Stressful Events Duration	0.46 **	—	0.12	0.06	0.16 *	−0.13	0.11	−0.02
3. Alcohol Use Frequency (# of days)	0.01	0.04	—	0.68 **	0.41 **	−0.05	0.10	0.08
4. Alcohol Use Amount (# of drinks)	0.12	0.10	0.72 **	—	0.57 **	−0.18 **	0.06	−0.10
5. Alcohol Use Severity (AUDIT)	0.15 *	0.14 *	0.58 **	0.67 **	—	−0.23 **	0.09	−0.24 **
6. Social Support	−0.13 *	−0.15 *	0.06	−0.05	−0.15 *	—	−0.24 **	0.22 **
7. Age	0.00	0.01	0.15 *	0.15 *	0.16 *	−0.12	—	0.02
8. Education	−0.11	−0.03	−0.02	−0.08	−0.12	0.15 *	0.03	—

* *p* < 0.05. ** *p* < 0.01. *** *p* < 0.001. # indicates quantity (number).

**Table 3:** Model F or χ^2^ values and significance.

	Probability of Monthly or More Alcohol Use ^t^		AUDIT		Amount of Alcohol Consumed (30 Days)	
	Event #	Event Duration	Event #	Event Duration	Event #	Event Duration
Age	6.20 *	5.20 *	0.26	0.13	4.11 *	3.98 *
Education	0.23	0.21	1.87	3.38	1.3	2.6
Sex	6.48 *	7.60 **	32.38 ***	32.15 ***	20.50 ***	18.98 ***
Recent Life Events	1.47	9.00 **	19.50 ***	10.45 **	7.23 **	1.96
Social Support	2.21	2.58	0.76	2.63	2.2	3.87 *
Sex × Recent Life Events	0.01	0.003	0.35	0.17	0.31	0.85
Sex × Social Support	0.02	0.09	<0.001	0.03	0.16	0.09
Recent Life Events × Social Support	0.94	0.01	1.19	0.94	4.20 *	3.57
Sex × Recent Life Events × Social Support	0.04	2.74	1.71	5.88 **	3.81	1.41
Model Value	17.01 *	26.19 **	6.61 ***	6.18 ***	4.91 ***	4.18 ***

* *p* < 0.05. ** *p* < 0.01. *** *p* < 0.001. ^t^ values presented are χ^2^. # indicates quantity (number).

## Data Availability

The data that support the findings of this study are available on request from the corresponding author. The data are not publicly available due to privacy and consent restrictions.

## Abbreviations

The following abbreviations are used in this manuscript:

SS	Social Support
AUDIT	Alcohol Use Disorder Identification Test
CAI	Cumulative Adversity Index
ISEL	Interpersonal Support Evaluation List
